# Tumor Microenvironment as a Regulator of Radiation Therapy: New Insights into Stromal-Mediated Radioresistance

**DOI:** 10.3390/cancers12102916

**Published:** 2020-10-11

**Authors:** Varintra E. Krisnawan, Jennifer A. Stanley, Julie K. Schwarz, David G. DeNardo

**Affiliations:** 1 Department of Medicine, Washington University School of Medicine, St. Louis, MO 63110, USA; varintra@wustl.edu; 2 Department of Pathology and Immunology, Washington University School of Medicine, St. Louis, MO 63110, USA; 3 Department of Radiation Oncology, Washington University School of Medicine, St. Louis, MO 63110, USA; jastanley@wustl.edu (J.A.S.); jschwarz@wustl.edu (J.K.S.); 4 Siteman Cancer Center, Washington University School of Medicine, St. Louis, MO 63110, USA; 5 Department of Cell Biology and Physiology, Washington University School of Medicine, St. Louis, MO 63110, USA

**Keywords:** stroma, cancer-associated fibroblast (CAF), extracellular matrix (ECM), cytokine/chemokine, growth factors, pro- and anti-tumor immune cells, immunomodulatory roles, radiotherapy dose fractionation, radioresistance, radiosensitivity

## Abstract

**Simple Summary:**

Cancer is multifaceted and consists of more than just a collection of mutated cells. These cancerous cells reside along with other non-mutated cells in an extracellular matrix which together make up the tumor microenvironment or tumor stroma. The composition of the tumor microenvironment plays an integral role in cancer initiation, progression, and response to treatments. In this review, we discuss how the tumor microenvironment regulates the response and resistance to radiation therapy and what targeted agents have been used to combat stromal-mediated radiation resistance.

**Abstract:**

A tumor is a complex “organ” composed of malignant cancer cells harboring genetic aberrations surrounded by a stroma comprised of non-malignant cells and an extracellular matrix. Considerable evidence has demonstrated that components of the genetically “normal” tumor stroma contribute to tumor progression and resistance to a wide array of treatment modalities, including radiotherapy. Cancer-associated fibroblasts can promote radioresistance through their secreted factors, contact-mediated signaling, downstream pro-survival signaling pathways, immunomodulatory effects, and cancer stem cell-generating role. The extracellular matrix can govern radiation responsiveness by influencing oxygen availability and controlling the stability and bioavailability of growth factors and cytokines. Immune status regarding the presence of pro- and anti-tumor immune cells can regulate how tumors respond to radiation therapy. Furthermore, stromal cells including endothelial cells and adipocytes can modulate radiosensitivity through their roles in angiogenesis and vasculogenesis, and their secreted adipokines, respectively. Thus, to successfully eradicate cancers, it is important to consider how tumor stroma components interact with and regulate the response to radiation. Detailed knowledge of these interactions will help build a preclinical rationale to support the use of stromal-targeting agents in combination with radiotherapy to increase radiosensitivity.

## 1. Introduction

The field of oncology has evolved from a malignant mutated cancer cell-centered view to the understanding of cancer as a complex “organ” composed of both malignant cells and diverse nonmalignant cellular and non-cellular components termed the tumor stroma or tumor microenvironment (TME) [1,2,3,4,5]. The concept of cancer as a disease focusing only on malignant tumor cells has been deemed inaccurate; in some cancers, stromal cells represent the majority of cell types, as is frequently seen in pancreatic and breast cancers [6]. These cellular stromal components often include activated cancer-associated fibroblasts (CAFs), leukocytes, and vascular cells, but they also sometimes include other adjacent normal tissue/cells such as non-transformed epithelia, adipose tissue, or neurons [1,2,3,4,5]. The non-cellular compartment of the tumor stroma comprises extracellular matrix (ECM) components like collagens, laminins, fibrinogen, elastin, and proteoglycan, and secreted factors such as cytokines, chemokines, and sequestered growth factors [1,2,3,4,5,6,7,8,9,10,11]. Accumulating evidence highly suggests that malignant cancer cells and the tumor stroma reciprocally communicate with and influence one another, but this relationship is complex and remains poorly understood. To treat cancer as a disease, we cannot single-mindedly focus on cancer cells with their autonomous genetic mutations; we need to simultaneously consider the TME because its interactions with tumor cells often contribute to disease initiation, progression, and treatment response [2,3,4,6,12].

Radiation therapy (RT) is a powerful anti-cancer therapeutic used to treat up to 50−60% of cancer patients [12,13]. The goal of RT is to target highly proliferative cancer cells while sparing normal tissue. The concept of dose fractionation—delivering small daily RT doses over several days—is designed to exploit cancer cells’ vulnerabilities in repairing DNA damage, leading to their demise, while giving normal healthy cells a chance to activate their DNA repair and cell cycle mechanisms [13,14,15,16]. Historically, radiobiology has utilized linear quadratic modeling to estimate the therapeutic treatment ratio, with increasing radiation toxicity to cancer cells while avoiding surrounding normal tissue. This “therapeutic ratio” is based on differences between the DNA damage and repair kinetics of cancer and normal cells. The linear-quadratic model utilizes the α and β parameters to describe the linear and quadratic portions of the cell survival curve, respectively, and experimental evidence suggests that these parameters and the α:β ratio differ widely across and even within some tumor types [17,18]. Classical modeling predicts that delivering small doses of radiation over the course of multiple treatments (i.e., conventional dose fractionation) can increase the therapeutic ratio compared to single-dose delivery, and early studies using small and large animal models confirmed these effects [17,18,19]. However, recent evidence has called into question whether small doses of radiation delivered over a protracted treatment course (conventional fractionation) are required to achieve these effects.

Standard of care for the majority of solid tumors requires 50 to 70 Gy total radiation dose delivered with conventionally fractionated schedules, most commonly utilizing 1.8 to 2 Gy per fraction. Over the past decade significant technologic advances in image-guided radiation, tumor tracking, beam intensity modulation, and beam shaping have facilitated the capacity to precisely deliver higher dose per fraction to the tumor while sparing larger volumes of surrounding normal structures. This concept of hypofractionation, or higher fractional doses of radiation over fewer total fractions and commonly delivered with stereotactic guidance via stereotactic body radiotherapy (SBRT) or stereotactic radiosurgery (SRS), has demonstrated safety and efficacy in many tumor types [20,21,22,23]. However, data also suggest that the clinical effects of hypofractionation are not solely due to differences in tumor and normal tissue DNA repair kinetics but also to the effects of radiotherapy on the TME. Although largely anecdotal, TME alterations can be demonstrated through the observation of “out-of-field” or abscopal responses to focused radiation as first described in the early 1950s [24]. Since then, additional work has demonstrated that the abscopal response is dependent on alterations in the immune system and the surrounding stromal tissue. Radiation can result in immune cell priming, neoantigen and cytokine release, modification of tumor vasculature, and alteration of the ECM, all of which have the potential to be optimized to enhance RT efficacy [25,26,27,28,29].

**Figure 1:**
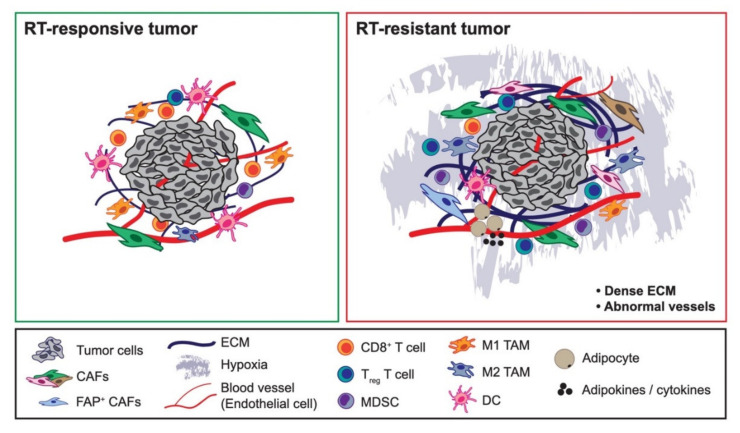
Components of the tumor microenvironment governing radiotherapy responsiveness. Components of the tumor stroma differentially dictate whether tumor cells are radiotherapy (RT)-responsive (Left) vs. RT-resistant (Right). Some tumor-promoting cancer-associated fibroblast (CAF) populations can cause tumors to be resistant to RT, such as fibroblast activated protein (FAP)^+^ CAFs. While immune cells such as CD8^+^ T cells, dendritic cells, and M1-like tumor-associated macrophages (TAMs) have been linked with RT-responsive tumors, pro-tumorigenic immune cells such as CD4^+^ T regulatory (T_reg_) cells, myeloid-derived suppressor cells, and M2-like TAMs have been associated with RT-resistant tumors. Dense extracellular matrix and abnormal endothelial cells and vessel formation, which contribute to tumor hypoxia, have been associated with RT-resistant tumors. Likewise, adipokines secreted by cancer-associated adipocytes can similarly cause tumors to be resistant to RT.

**Figure 2:**
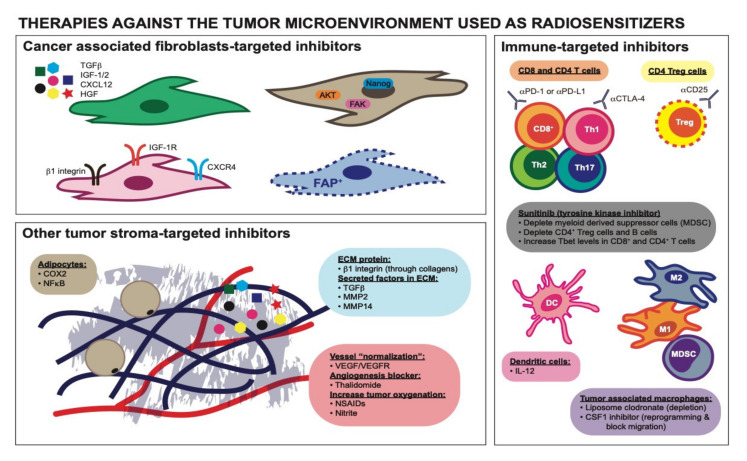
Therapies directed against distinct tumor stromal components used as radiosensitizers. Targeted inhibitors against CAFs’ secretory molecules, CAFs’ downstream cytosolic and nuclear signaling pathways, and CAFs’ receptors can increase RT efficacy. Targeting a unique population of CAFs, FAP^+^ CAFs, specifically for depletion can also radiosensitize tumors. Components of the ECM can activate β1 integrin receptor on CAFs and targeting β1 integrin can reverse tumor radioresistance. Targeted inhibitors against secreted factors reserved in the ECM have been shown to be radiosensitizing agents. “Normalizing” tumor vessels using VEGF/VEGFR inhibitors and Thalidomide can reverse tumor radioresistance. NSAIDs and nitrite have also been used as radiosensitizers due to their ability to increase tumor oxygenation. Inhibitors of COX-2 and NFκB targeted against cancer-associated adipocytes can reverse the radioresistance in tumors. Immune checkpoint inhibitors (αPD-1 or αPD-L1 and αCTLA-4 antibodies) can rescue “dysfunctional” CD8^+^ and/or CD4^+^ T cells and are beneficial when combined with RT. Depletion antibody αCD25 targeted against CD4^+^ T regulatory cells (T_regs_) can render tumor cells more radiosensitive. Interleukin-12 capable of enhancing the function of dendritic cells can increase the efficacy of RT. TAMs depletion agent, liposome clodronate, and CSF1 inhibitor can increase RT sensitivity. CSF1 inhibitor can also reprogram/polarize TAMs into having a more anti-tumorigenic phenotype. The tyrosine kinase inhibitor, sunitinib, has been used as a radiosensitizer due to its immunomodulatory ability.

Despite RT designed to target malignant tumor cells, and the knowledge that RT can be used to prime the immune system, the complex interaction between malignant tumor cells and other cells within the TME is important because the stroma can impact malignant cells’ response and contribute to treatment resistance [12]. Additionally, there are reports that RT can cause numerous changes in stromal cells within the TME that further promote undesirable tumor growth, invasion, and treatment resistance [12]. To successfully eradicate cancers, these reciprocal interactions between the tumor cells and tumor stroma must be characterized in detail. Moreover, the use of stromal-targeting agents in combination with RT is a largely unaddressed therapeutic option. These topics deserve more attention to broaden our knowledge to design better treatment strategies to combat cancers, particularly those characterized by a high density of stromal cells and other stromal components within the TME. In this review, we summarize the roles of stromal components and the TME that contribute to cancer cell radioresistance (Figure 1) and discuss how they may be targeted for possible therapeutic benefit (Figure 2).

## 2. The Impact of RT on the Tumor Stroma

RT seeks to exploit DNA repair deficiencies in malignant tumor cells, but even in perfect scenarios, it invariably affects stromal cells within the tumor mass or at its boundaries [30]. Although many RT-mediated stromal changes are beneficial, such as the revival of or polarization toward tumor-suppressing immunity, RT can act as a double-edged sword in tumors [13]. Specifically, deleterious side effects could facilitate cancer progression and treatment resistance. These are the byproducts of RT we hope to avoid by carefully designing and planning RT dose regimens and combinatorial treatments.

One impact of RT on the tumor stroma is chronic inflammation that drives RT-induced fibrosis marked by an increased number of stromal cells and ECM components, through several mechanisms that have been reviewed elsewhere [31]. RT-induced fibrosis is a well-known side effect that can arise in some patients [1,31,32]. RT can completely transform the TME by inducing rapid and chronic loss of hyaluronic acid [33] and collagen remodeling [34] by altering how CAFs regulate their collagen production [10,35]. Furthermore, RT-treated CAF populations can undergo modifications and alterations in terms of their diversity, secretome, and phenotype [36]. Additionally, RT enhances activation of proliferating machinery involving the RAS and mitogen-activated protein kinase (MAPK) cascades; the invasion pathways, which involve matrix metalloproteins (MMPs), laminin 5, and filamin A; transforming growth factor beta (TGFβ) signaling, which is involved in tumor progression, resistance, and metastasis [2,7,37]. Likewise, RT to the stroma can increase tumor invasiveness due to increased hepatocyte growth factor (HGF)/c-Met (HGF receptor) signaling and MAPK activity, which enhances tumor mobility and can be deleterious [38]. Taken together, these reports suggest that RT can activate stromal features indicative of potential stromal-mediated treatment resistance.

It is still unclear how different fractionated RT regimens alter the stromal components of the TME and how these changes affect subsequent responses of cancer cells to RT. Similarly, the attempts to combine stromal-disrupting agents with RT to overcome stromal-mediated radioresistance remain unclear and merit further study.

## 3. The Impact of CAFs on RT Efficacy

CAFs are heterogeneous and the major contributors to the tumor stroma [3,4]. CAFs have been shown to control tumor phenotype at all stages of tumor progression. Their roles have been reviewed elsewhere and include the ability to shape the ECM; modulate innate and adaptive immune microenvironments; recruit and regulate leukocyte migration and inflammation via cytokines, chemokines, and growth factors; provide metabolic support (amino acids, lipids, and tricarboxylic acid cycle intermediates); and contribute to paracrine activation of mitogenic and pro-survival cellular signaling via cell surface receptor-ligand interaction and secreted proteins or exosomes [3,4,39]. The mechanisms by which CAFs contribute to radioresistance are likely mediated through their secreted factors, contact-mediated signaling, immunomodulatory effects, and ECM alterations [40].

CAFs secrete a number of different active factors that have diverse effects on tumor phenotype [3,4]. For example, through their active secretory function, tumor stromal fibroblasts can transfer RNA within exosomes to cancer cells. This exosome transfer mechanism has been implicated in paracrine anti-viral RIG-I and juxtacrine NOTCH (NOTCH3-JAG1) signaling pathways, which both contributed to the expansion of therapy-resistant tumor-initiating cells. Stromal cells, which include CAFs and some bone marrow cells, protected breast cancer cells by inducing an interferon (IFN)-related DNA damage resistance signature in a STAT1-dependent manner and caused the tumors to be chemo- and radio-resistant [41]. In another model, conditioned media (CM) from pancreatic stellate cells (PSCs), which are the central mediator of desmoplasia and major contributors of pancreatic CAFs, dose-dependently enhanced pancreatic tumor cell proliferation, migration, invasion, and colony formation and caused resistance to gemcitabine and RT. The CM was found to activate the MAPK AKT pathways in tumor cells, and the authors postulated that factors such as interleukin-1β (IL-1β) and TGFβ were responsible [42]. The TME of glioblastoma is known to produce abundant TGFβ, a pleiotropic cytokine that promotes an effective DNA damage response. Glioma-initiating cells were protected from RT-induced cell death by this increase in TGFβ production, which promoted an effective DNA damage response and self-renewal via C-X-C chemokine receptor type 4 (CXCR4) and NOTCH1. TGFβ inhibition prevented tumor cell DNA repair and enhanced RT efficacy in this glioblastoma model [43]. Furthermore, CAFs can promote irradiated cancer cell recovery and tumor relapse after RT by producing insulin-like growth factor-1/2 (IGF-1/2), C-X-C motif chemokine ligand 12 (CXCL12), and β-hydroxybutyrate. These secreted factors increase reactive oxygen species (ROS) levels post-RT, which enhanced protein phosphatase 2A activity and repressed the mammalian target of rapamycin activation, therefore inducing autophagy in cancer cells to promote cancer cell recovery. It was argued that blocking IGF-2 and autophagy can reduce CAF-promoted tumor relapse in mice after RT and could be a promising RT sensitization therapeutic strategy (Figure 2) [44].

CAF-secreted factors trigger many downstream autocrine and/or paracrine signaling pathways that regulate treatment response. A network of paracrine signaling among cancer cells, myeloid cells, and stromal cells such as endothelial cells can drive the processes of treatment resistance and metastasis [45]. CXCL1 signaling is an example of paracrine signaling that contributes to radioresistance. Along with cancer cells, CAFs highly express and secrete CXCL1, which inhibits expression of the ROS-scavenging enzyme superoxide dismutase 1, leading to ROS accumulation following RT [46]. In this scenario, tumor cells take advantage of ROS accumulation to enhance DNA damage repair mechanisms and ultimately cause radioresistance. This radioresistance is also mediated by activation of the mitogen-activated protein kinase ERK kinase/extracellular-signal-regulated kinase (MEK/ERK) signaling pathway important for malignant transformation [47,48]. Crosstalk between CAFs and tumor cells through CXCL1 expression in an autocrine/paracrine signaling loop is responsible for the radioresistance phenotype [46]. Together, these studies showed that through their secreted active factors, CAFs can confer radioresistance to tumor cells.

CAFs are embedded in the tumor stroma, allowing them to actively communicate with other cells present in their surrounding environment through various mechanisms [3,4]. Besides acting through their secreted factors, CAFs also induce radioresistance through direct contact-mediated signaling between cancer cells and CAFs [3]. In pancreatic cancer, PSCs promote radioprotection and stimulate the proliferation of pancreatic cancer cells through β1 integrin signaling. β1 integrin is known to modulate the cellular response to genotoxic stress including RT [49]. It was found that this effect is independent of phosphoinositide 3-kinase (PI3K) but depends on focal adhesion kinase (FAK). β1 integrin inhibition or FAK knockout can abolish PSC-mediated radioprotection in pancreatic cancer cells to single-dose and fractionated RT [50]. These findings indicate that downstream cellular signaling pathways activated due to direct interactions between CAFs and tumor cells can mediate radioresistance.

Additionally, CAFs can work through paracrine networking to enrich cancer stem cells (CSCs), which have been implicated in chemo- and radioresistance. In pancreatic cancer, the presence of PSCs can induce CSC characteristics by increasing the epithelial-mesenchymal transition (EMT) phenotype. A proteomic screen revealed that TGFβ is involved in the radioresistance phenotype, and TGFβ neutralizing antibody can inhibit the EMT and CSC phenotype, thus sensitizing tumor cells to RT and reducing tumorigenicity in vivo [51]. In another setting, IGF-1 receptor (IGF-1R) signaling activation in cancer cells in the presence of CAFs expressing IGF-2 induced Oct3/4, Nanog, and Sox2 expression and promoted stemness pathways related to IGF-1R, EMT, PI3K, TGFβ, WNT, and Hedgehog signaling. This group showed that CAF-derived HGF, IGF-2, basic fibroblast growth factor (bFGF), WNT, and oncostatin M regulated CSC-like characteristics in a paracrine manner through counterpart receptor signaling components and stemness factors. They found that blocking IGF-2/IGF-1R/AKT/Nanog signaling reduced CSC stemness and concluded that there were potential clinical applications of targeted therapy to improve chemo- and radioresistance (Figure 2) [52]. Furthermore, CSCs play an important role in disease recurrence after RT as a result of their high DNA repair and antioxidative capabilities. Fractionated RT can enhance IGF-1 secretion and subsequent upregulation of IGF-1R in CSCs. IGF-1R upregulation exerts a dual radioprotective effect by inducing upregulation of AKT/ERK survival signaling and FoxO3 activation, which results in radiation protection. Additionally, they showed that inhibition of IGF-1R signaling reverses CSC radioresistance [53]. Collectively, these findings showed that CAFs can trigger radioresistance through their CSC-promoting roles.

In addition to CAFs’ de novo roles in mediating radioresistance, changes in CAFs due to RT can mediate further treatment resistance. This is important because most anti-cancer treatment regimens, including RT, are given in multiple treatment cycles with gaps to allow for normal cell recovery [32]. These gaps between treatment cycles can be exploited by both the tumor and stromal cells to take advantage of survival mechanisms. Thus, changes in the TME in between treatment cycles are important to consider with regard to the subsequent treatment response and resistance [32]. CAFs are not usually killed by RT; they are highly radioresistant due to the defective p53/p21 response pathway and high expression of the cancer marker Survivin [54]. Irradiated fibroblasts can promote the invasive growth of squamous cell carcinoma through the induction of c-Met, RAS, MAPK cascade (Raf-1, MEK1, ERK-1/2), MMP-1, MMP-9, laminin 5, and filamin A. Irradiated fibroblasts also express high levels of TGFβ1 [37]. The effects these irradiated fibroblasts can have on non-irradiated neighboring cells are referred to as radiation-induced bystander effects [55], and there is evidence that many of these factors can promote radioresistance.

There are several examples by which RT-induced changes in CAFs contribute to radioresistance. After genotoxic stress, CAFs can secrete WNT16B to the TME and promote prostate cancer therapy resistance. WNT16B, a secreted protein that is activated in fibroblasts through the nuclear factor (NF)κB pathway after DNA damage, subsequently activates the canonical WNT program and promotes EMT in neoplastic cells through paracrine signaling. This process attenuates the effects of both chemo- and radiotherapies and promotes tumor cell survival and disease progression [56]. Moreover, exposure to low-dose RT (<20 cGy) can induce premature senescence in stromal fibroblasts. In one setting, these senescent CAFs are responsible for stimulating enhanced proliferation of breast carcinoma cells and are correlated with radioresistance, which is partly mediated by the AKT pathway [57]. In addition, senescent CAFs can induce a senescence-associated secretory phenotype that includes the production of IL-6, IL-8, and osteopontin that are considered to be pro-tumorigenic factors and have been associated with immunosuppression and stromal-mediated therapeutic resistance [3,6,58]. RT can also promote EMT transition and invasion of pancreatic cancer cells by activating CAFs. CAF-derived CXCL12 directly promoted tumor cell EMT and invasion by acting through CXCR4 on tumor cells and downstream activation of the P38 pathway. Blocking CXCL12/CXCR4 signaling between pancreatic cancer cells and CAFs could attenuate RT-induced tumor cell invasion [59]. Indeed, HGF secretion by irradiated CAFs can increase phosphorylation of c-Met and MAPK activity in pancreatic tumor cells, which translates into enhanced invasion. This unwanted byproduct of RT can be overcome by blocking HGF signaling with an HGF antagonist [38]. Finally, exposure of CAFs to 18 Gy RT resulted in potent induction of multiple DNA damage response (DDR) foci; induced premature cellular senescence; and inhibited proliferative, migrative, and invasive capacity of CAFs. This RT dose increased the expression of integrins α2, β1, and α5 and dramatically augmented and redistributed focal contacts [60]. The increase in β1 integrin has been correlated with radioresistance [50]. All of these examples point to RT-induced changes in CAFs that can promote further radioresistance. This should encourage us to find therapeutic regimens that target both the tumor and stroma to successfully deliver anti-cancer treatment.

CAF-secreted factors, contact-mediated signaling, downstream pro-survival signaling pathways, CSC-generating role, and changes due to RT all comprise intricate crosstalk between CAFs and cancer cells to render tumor cells radioresistant. While we understand that CAFs play a critical role in shaping responses to RT, there are several areas where we do not fully understand their impact. First, CAFs in tumors are a diverse heterogeneous population that can have opposing roles [3,4,39]. While an abundance of literature supports the tumor-supporting roles of CAFs, some studies also suggest that certain CAF subsets may have tumor-restraining abilities [61,62]. These diverse CAF subsets may have differential effects on radioresistance and, in turn, be shaped differently by RT. Second, the plasticity, diverse origins, and spatial location of CAFs [40,63,64,65] may complicate things further in terms of their contributions to radioresistance. RT may alter CAF phenotypes temporally and spatially, and this will also affect cancer cells’ responses to RT. Lastly, RT dose fractionation may differentially impact CAF diversification. Our understanding of CAF diversity and plasticity is still limited, but it is logical to assume that CAF-mediated radioresistance is a problem in RT success and needs further study.

## 4. The Impact of ECM on RT Efficacy

The ECM plays an essential role in regulating cancer progression and radiosensitivity. Tumor ECMs are dynamic structures that are remodeled during tumor progression and/or treatment [8]. Among the approximately 300 proteins present in the ECM that are known to regulate tissue homeostasis, inflammation, and disease, collagen is the most abundant, constituting up to 90% of the tumor ECM. In addition to collagens, other prominent fibrous proteins are elastins, fibronectins, and laminins, which are also involved in controlling tumor phenotype [8]. Tumor ECM is typically denser and mechanically stiffer than normal ECM, due to the quantity of ECM as well as structural changes in molecular architecture such as the extent of crosslinking [66]. These changes in ECM density, composition, and stiffness significantly impact malignant cell invasion, survival, and proliferation [67,68]. Variations in ECM stiffness and density have been correlated with disease aggressiveness, progression-free survival, and in some cases, resistance to different treatment modalities [8].

The ability of tumor-promoting ECM to drive treatment resistance is particularly applicable to RT. Tumor cells can interact with the ECM through direct interaction (cell-protein contact), and one major way is through the engagement with integrins [49]. ECM stiffness can facilitate integrin clustering, which can lead to activation of downstream FAK activation and MAP/ERK kinase signaling pathways leading to cell survival, proliferation, migration, and invasion. Integrin-mediated adhesions can also activate transcription factors NFκB, inositol lipid metabolism, and MMP activity [49], in addition to the activation of PI3K/AKT and RAS/MAPK pathways. Integrin activation is important in regulating tumor phenotypes and has been associated with processes such as angiogenesis, survival, invasion, metastasis, and treatment resistance [2,4]. Further complicating this is the fact that RT can increase the expression of integrins α2, α5, β1, and β6; therefore, we need to consider how RT fractionation controls subsequent treatment resistance [60,69,70]. For example, it was found that β1 integrin controls radioresistance by resisting cellular apoptosis from RT through the activation of AKT signaling. Inhibition of β1 integrin can resensitize tumor cells to RT by decreasing proliferation and increasing apoptosis (Figure 2) [71,72]. Another group also found that β1 integrin-mediated adhesion confers RT resistance through downstream FAK-interacting proteins (p130Cas and paxillin) and PI3K/AKT-mediated pro-survival signaling pathways [73]. Together, these examples showed that contact-mediated signaling between tumor cells and ECM proteins in cancers can contribute to radioresistance.

The ECM also contains secreted soluble signaling molecules from tumor and stromal cells. The ECM acts as a reservoir for these cytokines, chemokines, and growth factors. Two prominent examples of these secreted factors that control tumor phenotype are TGFβ and various members of the MMP family. Besides interacting directly with the stromal cells and surface proteins in the ECM, tumor cells are constantly and sophisticatedly communicating with these secreted regulatory molecules [10]. Many of these proteins are already known to be capable of inducing radioresistance, such as TGFβ [42,43,51]. Moreover, RT can further increase TGFβ levels, which can accelerate tumor progression. Inhibition with TGFβ neutralizing antibodies has been shown to prevent radiation-induced metastatic progression [74]. Another class of secreted proteins that are highly abundant in the ECM and mediate tumor progression are the different MMPs [2,8,75]. MMP2 is known to degrade collagen IV and plays a role in RT-induced lung injury. MMP2 inhibition prior to RT abrogated the induction of FoxM1 expression, reduced p53 and p21 expression, decreased expression of DNA repair genes XRCC1 and Chk2/1, and abrogated G2 cell cycle arrest, leading to apoptosis and enhanced radiosensitivity [76]. These examples showed that the ECM serves as a tumor growth factor and cytokine sink, which contributes to tumor radioresistance and worthy of consideration in future therapeutic planning.

Besides controlling the stability and bioavailability of numerous growth factors and cytokines, ECM structure and integrity also influence oxygen availability, acidity, and interstitial fluid pressure in tumors, through its regulation of the tumor vascular system, so it has important effects in terms of controlling treatment response (Figure 1) [8,66]. Oxygen availability is critical for RT response as hypoxic cells are generally 2.5−3 times less radiosensitive than normoxic cells [77,78,79,80]. The indirect effects of RT produce ROS through the hydrolysis of water, which then propagate and modify lipids, membranes, and proteins [10]. Using nitric oxide-dependent arteriole vasorelaxation as a way to increase the partial pressure of oxygen in tumors, multiple groups found that low-dose nitrite can sensitize tumors to RT, leading to a significant tumor growth delay and longer survival [81,82]. Antiangiogenic therapy such as vascular endothelial growth factor (VEGF) receptor 2 blockade, which can create a “normalization window” that increases tumor oxygenation, has also been shown to enhance the RT response (Figure 2). This effect is dependent on the increased pericyte coverage of tumor vessels via the upregulation of angiopoietin 1 and degradation of the pathologically thick basement membrane via MMP activation [83]. Thalidomide, an angiogenesis inhibitor, can also increase tumor reoxygenation correlated with reduced interstitial fluid pressure and increased perfusion, sufficient to radiosensitize tumors [84]. Non-steroidal anti-inflammatory drugs (NSAIDs) are another radiosensitizing drug class that works through increasing tumor oxygenation via either a decrease in macrophage recruitment or inhibiting mitochondrial respiration. Using four different NSAIDs (diclofenac, indomethacin, piroxicam, and NS-398), radiation sensitivity in tumor cells can be increased by enhancing radioinduced apoptosis and inhibiting repair of sublethal RT damage [85]. These studies showed that tumor ECM governs oxygen bioavailability in cancers, controls radiosensitivity, and that RT requires sufficient tumor oxygenation to avoid radioresistance (Figure 1).

In most cancers, tumor cells are embedded in stromal cells with abundant ECM components, that, as described above, govern radioresistance through direct interaction with tumor cells and the ECM’s roles as protein reservoirs and a major controller of tumor oxygen bioavailability. Even though many of these factors negatively impact RT efficacy, there are known inhibitors that can successfully reverse ECM-mediated radioresistance. As with CAFs, our current knowledge is still lacking on how RT dose fractionation may differentially impact ECM alterations and how they contribute to radioresistance.

## 5. The Impact of Immune Cells on RT Efficacy

RT is a powerful therapeutic approach used in many patients due to its numerous beneficial effects leading to tumor cell eradication. Besides the direct killing of highly proliferating tumor cells by mitotic catastrophe or apoptosis or necrosis, RT is increasingly appreciated to have immunomodulatory effects, which can take advantage of the fact that our immune cells can target and kill abnormal cancerous cells [11,13,30,32,86]. RT modulates the immunogenicity and adjuvanticity of tumors by increasing the expression and release of tumor-associated antigens, increasing the expression of major histocompatibility complex I (MHC-I), inducing immunogenic cell death (ICD) and its downstream anti-tumor pathways, and releasing danger signals and chemokines that recruit inflammatory anti-tumor immune cells to the TME, including antigen-presenting cells that can activate cytolytic T cells [75,87,88]. RT can also enhance tumor killing by increasing the number of tumor-infiltrating immunostimulatory cells and neoantigen expression [89,90,91]. However, there are reports that RT can induce immunosuppression on top of anti-tumor immune promoting effects [12,32]. The balance between the two variables predicts the treatment response. Due to its double-edged sword effects on immune modulation, precise dosing regimens and combinatorial treatments of RT must be carefully considered to avoid unwanted immunosuppressive effects [7,9,10,11,13,30,32,86,87].

As we have discussed with CAFs and ECM, radiosensitivity depends on the complex interaction of malignant cancer cells with their immune TME [86]. Notably, the host immune status also determines the efficacy of treatments, including RT (Figure 1). The presence and activation status of dendritic cells (DCs) and CD8^+^ T cells and anti-tumor cytokines such as IFNγ determine the responsiveness to RT [92,93,94,95]. IFN-related DNA damage resistance signature (IRDS) genes including STAT1, IFN-stimulated genes 15 (ISG15), and IFN-induced protein with tetratricopeptide repeats 1 (IFIT1) are associated with resistance to chemotherapy and/or RT across different tumor cell lines [69]. Additionally, the intratumoral immune response after RT also determines the therapeutic response. RT success is dependent on the antigen-specific nature of immune activation, which can be enhanced by combining RT with immune checkpoint blockade therapies like αPD-1 and αCTLA-4 [26,87,96,97]. Increases in intratumoral anti-tumor immune cells and IFNγ were found to imbue CD8^+^ T cells with lytic activity against tumor cells, and the addition of IL-12 as immunotherapy can augment this RT-induced anti-tumor immunity [94,98]. Radiation can upregulate the expression of programmed death ligand 1 (PD-L1) on tumor cells in numerous in vivo models, which binds with immune checkpoint receptor PD-1 expressed by CTLs and thereby promotes their dysfunction. The combination of radiation and inhibition of this immune checkpoint has been shown to improve the radiation-induced anti-tumor response through activation of cytotoxic T cells and diminished the influx of myeloid-derived suppressive cells (MDSCs) into the TME [99,100,101]. Interestingly, several studies in mice and humans have demonstrated abscopal effects, with response of both the primary tumor and distant disease to combination therapy of RT and immune checkpoint blockade [102]. Although most of these studies were conducted utilizing hypofractionated or single-dose ablative radiation schemes, the optimal dose, fractionation, and timing to achieve this effect are unknown [103]. In vivo studies have demonstrated that PD-L1 expression peaks at 3 days post-irradiation, and that concurrent but not sequential treatment with checkpoint inhibitors is necessary for a T cell-mediated tumor response [93,101]. Interestingly, in the KEYNOTE-001 trial, patients who received radiation at any time prior to immune checkpoint therapy had significantly increased progression-free survival, with median time ranging from 9.5 to 11.5 months [104,105]. Despite these appealing data, larger studies demonstrating sufficient survival benefits to lead to approval of regimens containing RT in combination with immune checkpoints have not been published. However, these findings showed the importance of the host’s immune status as one major contributor that can predict radiosensitivity.

Like CAFs, immune cells in the TME are very diverse and have different roles. Tumor-associated macrophages (TAMs), MDSCs, and CD4^+^ regulatory T cells (T_regs_) are known to be immune suppressive and pro-tumorigenic; on the other hand, immune cells such as DCs, CD8^+^ T cells, and NK cells are anti-tumorigenic [12]. Likewise, these immune cells also differentially regulate radiosensitivity in cancers (Figure 1). The presence of TAMs correlates with increased radioresistance in many different tumors [106]. Radiation plays a role in the recruitment and phenotype modulation of TAMs in the TME. Recruitment of TAMs occurs irrespective of dose and fractionation [107]. Conventional fractionation leads to transcription of colony-stimulating factor 1 (CSF1), which when blocked in prostate cancer models reduces TAM recruitment. Conversely, hypofractionated regimens promote TAM recruitment in hypoxic conditions, where in glioma models radiation-induced hypoxia-inducible factor 1 (HIF-1) expression leads to the increased density of TAMs [108,109]. Co-implantation of tumor cells with bone marrow-derived macrophages increased tumor radioresistance, so depletion of TAMs using a systemic or local injection of macrophage-depleting liposomal clodronate before RT can increase anti-tumor effects in different RT dosing regimens. Radioresistance coming from TAMs is mediated by the tumor necrosis factor (TNF) signaling-dependent upregulation of VEGF, and anti-VEGF or anti-TNF therapy can reverse this radioresistance (Figure 2) [110]. The effect of the radiation-induced influx of TAMs in the TME depends on the exact phenotype they acquire once infiltrated. The decision tree for macrophage polarization, unlike recruitment, does appear to be dependent on the radiation dose and fraction. Anti-tumor phenotypes may be favored in conventionally fractionated dosing, which can in turn enhance T cell-mediated tumor control [111,112]. Importantly, this effect may be lost in hypofractionated and single-dose ablative regimens. One reason may be that hypoxia which results from vascular impairment by RT promotes macrophage immunosuppressive and pro-tumorigenic tissue remodeling functions [113,114,115]. However, this is likely an oversimplification, and the true impact of TAMs may depend on the organ- and cancer-specific context in which radiation is employed. RT itself can cause an influx of MDSCs into tumors that eventually polarize the TME into an immune-suppressive environment. This polarization is dependent on transcriptional regulation by the NFκB p50 subunit, as mice lacking NFκB p50 are much more sensitive to RT [116]. Additionally, MDSCs can be recruited further into the TME from RT in a fractionated RT regimen due to the recruitment of DNA damage-induced kinase ABL1 into cell nuclei where it binds the CSF1 gene promoter and enhances its transcription. Hence, blocking macrophage migration with a CSF1 inhibitor radiosensitizes tumors [46]. Another group also found that a neutralizing antibody to CSF1 or a small molecular inhibitor to the CSF1 receptor kinase efficiently depletes macrophages and delays tumor regrowth following RT (Figure 2). This delay is a reflection of the increased presence of CD8^+^ T cells and reduced presence of CD4^+^ T cells, the main source of T helper 2 (Th2) cytokines IL-4 and IL-13. The authors proposed that the response to RT could be enhanced by reducing TAMs in tumors or blocking their induction of Th2 polarization [117]. Moreover, treatment with the small tyrosine kinase inhibitor sunitinib resulted in a significant reduction of MDSCs and phospho-STAT3 and increased T cell proliferative activity in cancer patients. Sunitinib’s ability to increase RT efficacy in tumors is mediated through the reduction in the number and function of immunosuppressive MDSCs and is significantly correlated with lower CD4^+^ T_reg_ and B cell numbers and augmentation of Tbet expression in primary CD4^+^ and CD8^+^ T cells [118,119]. Finally, due to the intrinsic radioresistant nature of CD4^+^ T_regs_ and its immunosuppressive roles, CD4^+^ T_reg_ presence in tumors has also been correlated with radioresistance [120,121]. T_regs_ are essential to generating immune tolerance, are radioresistant compared to other T cell subtypes, and demonstrate a relative increase in the TME after irradiation. T_regs_ are known to increase in mice receiving whole-body radiation [122], and studies of human cervical cancers treated with 10-30 Gy demonstrate decreased CD8^+^ and CD4^+^ T cells, without any effect on T_reg_ numbers [123]. In vivo tumor models with systemically depleted T_regs_ demonstrate significant determent of primary and metastatic tumor progression. When these tumors are irradiated, these models demonstrate significantly reduced tumor burden post-RT and improved overall survival [124,125,126,127,128,129]. Dose and fractionation have also been demonstrated to play a role in the balance of immune priming and immunosuppression post-RT. A B16 murine model receiving a single 5 Gy dose of irradiation showed a relative increase in the T_reg_ population compared to cytotoxic T cells. However, a single 10 Gy dose resulted in a relative decrease of T_regs_, while a single 15 Gy dose increased both T_reg_ and effector T cells [130]. Systemic elimination of CD4^+^ T_regs_ using anti-CD25 monoclonal antibody enhances radiotherapeutic benefits via immune modulation (Figure 2) [122]. Collectively, these studies showed that pro-tumorigenic immune cells can modulate radioresistance in many cancers.

While initially thought to create an immunosuppressive TME, radiation has recently gained momentum clinically as a means to prime the immune system to recognize and remove tumor cells. To achieve this end, cross-presentation of tumor antigen by DCs to cytotoxic T cells must be upregulated. Radiation has been shown to increase IFN-I signaling, leading to expansion and activation of DCs, through induction of the stimulator of IFN genes (STING) pathway [26,93,131,132]. Additionally, tumor cell removal requires the induction of cell death pathways, which can be variable, and include apoptosis, necrosis, autophagy, and mitotic catastrophe. Immunogenic cell death upregulation is frequently observed following irradiation. This involves three key molecular signals: calreticulin, which undergoes translocation from the endoplasmic reticulum to the plasma membrane to signal uptake of dying tumor cells by DCs; high-mobility group protein B1 (HMGB1), which is released from the dying cells to bind Toll-like receptor 4 on DCs promoting antigen cross-presentation; and adenosine triphosphate (ATP), which activates cytotoxic T cells through inflammasome activation via the P2XR7 pathway [86,132,133,134,135]. In vitro experiments have demonstrated that the generation of these three key signaling molecules is dependent on irradiation dose [88]. However, the optimal dose and fractionation of irradiation required to induce ICD in vivo is influenced by the TME [75,86,133]. Utilizing a B16 melanoma mouse model expressing ovalbumin antigen, several groups have demonstrated that single-dose, 15-20 Gy irradiation was more effective in generating activated cytotoxic T cells than more conventional fractionated schedules of 15 Gy in 5 daily fractions and 20 Gy in 4 bi-weekly fractions. This indicated that the prescribed dose per fraction and also the specific timing of individual dose delivery are important to elicit RT-induced immune responses [26,131]. Clinical investigations of this mechanism in patients with colorectal and prostate cancer demonstrated a detectable increase in circulating cytotoxic T cells (Survivin- and/or prostate-specific antigen-specific) in post-irradiation blood samples [136,137]. Additionally, early-stage non-small cell lung cancer patients treated with SBRT, 48 Gy in 6-8 fractions, showed increased circulating cytotoxic T cells [138]. Many studies have indicated that ablative radiation doses are required for activation of T cell immunity, and this is corroborated by clinical evidence suggesting that conventional fractionation can have a detrimental effect on the TME as a result of the death of infiltrating anti-tumor lymphocytes [32,139,140,141]. In preclinical studies, mice bearing bilateral flank implants of CT26 colon carcinoma treated with conventional fractionation (10 Gy in 5 fractions) initially demonstrated T cell reductions after each dose of radiation; however, this regimen at 7 days post-therapy led to an expansion of local polyclonal T cells responses and infiltrating T cells, revealing a treatment duration effect in this model system [142]. Several groups have moved away from flank models, due to the prevailing theory that to initiate tumor growth you create a wound stimulating a subsequent acute immune response. Instead, several groups have adopted genetically-engineered mouse models (GEMMs) that spontaneously form tumors. Recent publications utilizing GEMMs of pancreatic cancer and sarcoma have demonstrated that reprogramming the TME can induce T cell immunity and sensitize tumors to radiation [143,144,145] from both single high-dose and hypofractionated RT schemes.

Radiation also plays an important role in overcoming T cell exclusion from the TME. One barrier is the dampened homing of effector T cells, which is modulated by cytokines released from tumor cells and the surrounding stroma. Irradiation can significantly enhance the secretion of CXCL16 by mouse and human breast cancer cells; this chemokine binds to CXCR6 on activated cytotoxic T cells and plays an important role in their recruitment to inflammation sites. CXCL16 can be induced in vitro by a single fraction dose of 5 Gy; while in vivo induction was found to be dose-dependent, reaching a plateau at 12 Gy [146,147]. In another study, mice deficient in IFNγ, a cytokine that is critical for innate and adaptive immunity, received tumor-localized irradiation and demonstrated decreased expression of MHC-I and CXCL9/CXCL10, which are important chemoattractants for cytotoxic T cells [147,148]. TNFα can also be induced by single-fraction irradiation in tumor cell lines [149]. Radiation also leads to upregulation of anti-inflammatory cytokines, like TGFβ, which will suppress the function of DCs and cytotoxic T cells and promote maturation of T_regs_. Single fraction radiation from 5–10 Gy in mouse mammary tumors upregulated TGFβ in both tumor cells and the surrounding adipose stroma [34,150]. In irradiated tissues, there is a relative increase in ROS, leading to activation of TGFβ. Mouse models of mammary carcinoma demonstrate that activation of DCs and priming of cytotoxic T cells during RT can be improved by administering TGFβ-neutralizing antibodies [99,150].

Tumors counteract this influx of activated cytotoxic T cells through the downregulation of antigen-presenting MHC-I proteins. Historical studies revealed that radiation increases MHC-I protein expression on tumor cells, leading to restored antigen recognition by cytotoxic T cells. In primary glioblastoma lines, increasing doses of radiation up to 12 Gy led to increased MHC-I expression, and a similar effect was demonstrated in ovarian and cervical cancer cell lines with doses of 25 to 100 Gy [151,152,153,154]. Conventionally fractionated radiation also can induce MHC-I expression, where conditioned media from breast cancer lines treated with 6-10 Gy delivered in 3-5 fractions was able to stimulate expression of total cellular and surface MHC-I in recipient cells [155].

The interactions between tumor cells and their immune microenvironment is very complex due to their abundance, diversity, and varying roles. Evidence showing the presence of anti-tumor immune cells and IRDS genes being important to determine RT efficacy and the roles of pro-tumor immune cells to promote radioresistance should inspire us to design therapeutic regimens with different immune-modulating drugs to synergize with RT. However, it is still unclear how different RT fractions affect immune TME. Additionally, it was recently discovered that distinct immune cell origins (bone marrow or embryonic) have different roles in controlling tumor phenotype [156]. How the immune cell origin contributes to immune-mediated radioresistance is still a largely unaddressed question in the field and needs to be studied [157,158].

## 6. Immunomodulatory Roles of CAFs and the ECM and How Their Interactions Regulate RT Efficacy

With recent discoveries, it is increasingly appreciated that malignant tumor cells interact with their TME in a complex and reciprocal manner to regulate tumor progression and treatment response [1,2,3,4,5]. In addition to the ability of the individual stromal components to directly control tumor cell phenotype, interactions among the components of the TME themselves can also exert the same effects [6,66]. Immune cells in the TME are regulated by their microenvironment, including the CAFs and ECM in addition to the tumor cells. These communal interactions in the TME are important to consider because they can inadvertently affect treatment response [11,32,86].

CAFs have various pro-tumorigenic roles by regulating tumor immunity, ECM, and hypoxia, among many other factors [66]. One group reported that fibroblast activation protein (FAP)^+^ CAFs, one subtype of CAFs, are responsible for suppressing anti-tumor immunity and thus contribute to uncontrollable tumor growth. Depleting FAP-expressing cells provides some tumor growth control through a process involving IFNγ and TNFα [159]. In a cervical cancer model, mesenchymal stromal cells were responsible for immunosuppression through their ability to dampen CD8^+^ T cell proliferation, activation, and effector functions. This was found to be mediated by the expression of CD39 and CD73 ectonucleotidases and the generation of adenosine by the stromal cells [160]. Lastly, CAFs can support tumorigenesis and mediate tumor-enhancing inflammation by enhancing tumor angiogenesis, proliferation, and invasion. These tumor-promoting characteristics are mediated by NFκB pathways [161]. These tumor-promoting roles of CAFs, through their modulation of tumor immunity, may apply to the mechanisms of stromal-mediated radioresistance.

CAFs can control tumor immunity through multiple mechanisms [66,162]. CAFs regulate both adaptive and innate immune cell functions, including T cell and immunosuppressive myeloid cells. CAFs can negatively impair the function of CD8^+^ T cells including cytolytic activity and cytokine production through the production of soluble factors, such as TGFβ and VEGF, metabolic reprogramming via indoleamine 2,3-dioxygenase and arginase, and expression of checkpoint inhibitors like PD-L1 [66,163]. Many of these factors were discussed previously in the other sections as they are implicated in radioresistance [42,43,51,110]. CAFs can also affect myeloid cell and DC maturation status and function [164,165,166,167]. These immune cells are essential mediators of radiosensitivity [92,93,94,95,106]. More importantly, many recent discoveries have shown how targeting the stroma can reawaken anti-tumor immunity and synergize with immunotherapy [168,169,170], as reviewed elsewhere [66]. The concept of how stromal interactions with the immune microenvironment affect immunotherapy response may also hold true for RT responses.

One way by which CAFs regulate the immune TME is through their secreted factors. CAFs secrete many active factors to the TME which are eventually stored in the ECM reservoir, including the various MMPs [2,8,10,75]. MMP14 mediates tumor progression through vascular and immune-modulatory effects. The anti-MMP14 inhibitory antibody can inhibit tumor growth, reduce tissue hypoxia, increase macrophage number, and shift cell phenotype towards the more anti-tumor M1-like phenotype due to reduced active TGFβ and SMAD2/3 signaling, hence synergistically enhancing RT effects (Figure 2) [171]. This example demonstrates that CAF interactions with the ECM and the immune microenvironment can regulate tumor cell radiosensitivity.

CAFs and the ECM have many immunomodulatory functions, which can control tumor cells’ treatment response, including RT efficacy. Even on their own, CAFs, ECM, and immune cells can directly confer a radioresistance phenotype to tumor cells, and their interactions among themselves also affect how tumor cells respond to RT. It is presently unclear how these relationships among the tumor stroma components are affected by host immune status and vice versa. Similarly, it is not known how different RT doses and fractionations change these complex interactions and their ensuing tumor-regulating phenotype. It will be fascinating to see how new technologies such as single-cell RNA sequencing will help us discover novel stromal and immune cells and shape our understanding of the immunomodulatory functions of stromal cells in cancers.

## 7. The Impact of Other Stromal Cells on RT Efficacy

The TME contains other stromal cells present besides CAFs: blood endothelial cells, lymphatic endothelial cells, adipocytes, mesenchymal stem cells, fibrocytes, pericytes, neurons, etc. Although their contribution is small in terms of their numbers in the TME, they still play important roles in tumor progression, treatment response, treatment resistance, and cancer metastasis [1,7].

Endothelial cells are important players in many different types of cancer. They supply nutrients for tumor growth, provide routes for metastatic dissemination, and contribute to chemo- and radioresistance [80,172,173]. Hence, it is important to understand the tumor vasculature to comprehend endothelial cell-mediated radioresistance. Tumors have two main ways to develop vasculature: angiogenesis and vasculogenesis. Angiogenesis is the process in which vessels are developed from nearby endothelial cells, while vasculogenesis is the formation of blood vessels from circulating cells postulated to come from the bone marrow [80]. It is clear that angiogenesis is an important process fundamental to treatment refractoriness, but vasculogenesis is especially imperative in RT resistance due to the fact that local RT abrogates local angiogenesis, forcing tumors to rely heavily on the vasculogenesis pathway for blood vessel regrowth post-RT. This mechanism poses another barrier to T cell infiltration. Dysfunctional tumor-associated vasculature has endothelial cells lining the vessels that suppress T cell activity, target them for destruction, and block entry into the TME [174]. Studies of ablative doses of radiation as high as 25 Gy led to the infiltration of TAMs expressing immunosuppressive enzymes [175]. Notably, single-fraction ablative doses also induce significant endothelial cell death, causing reduced vascular flow, hampering T cell recruitment, and inducing a hypoxic and immunosuppressive TME [176,177]. These ablative doses have been evaluated by bioinformatic studies, demonstrating that alteration of tumor vasculature post-irradiation accounts for 20−30% of the radiographic response of brain metastases in stereotactic radiosurgery cases [178]. In contrast, radiation doses <10 Gy have been shown to promote vascular relaxation and increase tumor oxygenation, with fractionated schedules providing the maximal benefit on tumor growth delay due to tumor reoxygenation [179,180]. Low-dose radiation also plays a role in the reprogramming of macrophages, which are important during the angiogenesis process, allowing increased T cell extravasation in vivo through inducible nitric oxide synthetase [112]. There is an influx of CD11b^+^ myeloid cells following tumor irradiation, and increased tumor hypoxia increases HIF-1 levels and subsequently upregulates stromal cell-derived factor 1 (SDF1, also known as CXCL12) to initiate vasculogenesis [80]. Many angiogenic inhibitors have been used in the clinic and have proven efficacy as radiosensitizing agents because of multiple different mechanisms related to the “normalization” of tumor vessels and their subsequent oxygen content and acidity (Figure 2) [2,6,32]. One group claims that the use of the VEGF receptor inhibitor axitinib radiosensitized tumor endothelium and enabled tumor control [181]. Another group found that angiogenesis-promoting factors that protect against endothelial damage can diminish the RT response. Reversing this effect with a VEGF inhibitor promotes RT-induced endothelial injury through the generation of the second messenger ceramide through acid sphingomyelinase trafficking to the plasma membrane. This tumor endothelial cell RT resensitization was shown to mediate tumor control [181]. These studies demonstrated that endothelial cells are important mediators of tumor radioresistance due to their ability to form vessels and control tumor oxygen content.

Adipocytes are also active players in the TME that control cancer development, progression, metastasis, and treatment response, especially in cancers that interact closely with adipose tissue-like breast cancers [182]. Cancer-associated adipocytes (CAAs) are energetic cells capable of secreting a heterogeneous group of molecules known as adipokines that include hormones, growth factors, and cytokines. Some examples of adipokines are leptin, adiponectin, autotaxin, IL-6, TNFα, IGF-1, and HGF. In addition, CAAs actively participate in metabolic remodeling that supports cancer cell growth by regulating the fatty acid reservoir to increase mitochondrial β-oxidation. They also interact closely with CAFs and ECM molecules through ECM remodeling. More importantly, they act as obstacles to various anti-cancer therapies as they are involved in diverse resistance mechanisms [1,7].

One mechanism of adipocyte-mediated radioresistance is through their secreted adipokines. Some of these adipokines such as TNFα, IGF-1, and HGF were discussed earlier with regard to their contributions to stromal-mediated radioresistance (Figure 1) [38,52,110,159]. Another group reported that these adipokines can increase the gene expression of NFκB and cyclin D to induce anti-apoptotic transcription and stabilize pro-oncogenic factors such as β-catenin and cyclin-dependent kinases [182]. It was also found that breast cancer cells co-cultured with adipocytes are radioresistant, and the mechanism is through adipocyte secretion of IL-6 resulting in the phosphorylation of Chk1 associated with decreased cancer cell death [182,183]. Another mechanism of adipocyte-mediated radioresistance is the initiation of autotaxin (ATX)–lysophosphatidic acid (LPA) signaling. This group found that CAAs, which closely interact with adjacent tumor cells, can become inflamed from tumor-derived cytokines, which results in the stimulation of adipocytes’ ATX secretion and subsequent LPA production. This further promotes inflammatory cytokine production in a vicious feed-forward cycle. RT-induced adipocyte injury triggers increased levels of ATX, cyclooxygenase-2 (COX-2), IL-1β, IL-6, IL-10, TNFα, and LPA1 and LPA2 receptors. This inflammatory response depends on the DNA damage response pathways ATM, ATR, and PARP-1 and inflammatory mediators COX-2 and NFκB, which can potentially be inhibited to reverse radioresistance. Induction of LPA signaling enhances lymphocyte invasion and cytokine and VEGF production to stimulate angiogenesis required for tumor growth [184]. As detailed earlier, angiogenesis is one factor that contributes to tumor cell radioresistance [8]. These studies showed that adipocytes are not dormant “fat” cells; they are active contributors to tumor phenotype through their active metabolism and secreted factors.

Even though endothelial cells and adipocytes do not make up a significant portion of the tumor stroma, they contribute to tumor radioresistance. Multiple groups have shown that we should not negate their roles in controlling RT response to prevent the formation of radioresistant cancer cells. However, the complexity of different RT doses and fractionation regimens has left the field with an unanswered question regarding their effect on stromal cells present in cancers.

## 8. Forward Looking Conclusions

Malignant tumor cells that harbor genetic aberrations are in close proximity with the tumor stroma composed of diverse cellular and non-cellular entities including CAFs, ECM components, immune cells, endothelial cells, adipocytes, and secreted bioactive molecules. At every step of the tumor lifecycle, there is reciprocal communication between these malignant cancer cells and their neighbors. Their highly dynamic interactions control tumor initiation, progression, invasion, metastasis, and treatment resistance, which complicates cancer therapeutic planning [1,2,3,4,5,6,8,10,11,12,13,185]. Here, we reviewed how the tumor stroma can contribute to cancer radioresistance; many of which are mediated through secreted factors, cell surface receptors, and downstream pro-survival and/or anti-apoptotic signaling pathways. The tumor stroma is very diverse, and our current knowledge of distinct cell types is still lacking. It will be interesting to see how new technologies to discover novel CAFs and immune cell types will broaden our knowledge of tumor-stromal interactions.

Further complicating the concept of stromal-mediated radioresistance is the fact that with successive treatments tumor and stromal cells can become more resistant, which could be a problem for many anti-cancer treatment regimens given in cycles, such as chemo- and radiotherapies. Based on the potential of these tumor stroma to induce radioresistance, it seems plausible to include stromal-targeted agents in combination with RT for therapeutic benefit. Multi-directed treatments toward both the tumor cells and the tumor stroma could help eradicate cancers and prevent therapeutic resistance and tumor relapse.

Lastly, RT is a rapidly evolving field. Ultra-high dose rate of RT (FLASH-RT) is a new technology that enables the ultra-fast delivery of doses while sparing normal tissues [186,187]. It will be important to see how FLASH-RT influences tumor-stroma communications and stromal-mediated radioresistance. Despite many recent discoveries, there are still many remaining questions in the field to be addressed, such as how CAF diversity affects radioresistance and how the tumor stroma changes with different RT dosing and fractionation regimens. Future discoveries about these mechanisms can be used for the design of novel RT and drug combinations to target stromal-mediated RT resistance.
